# Material–Structural Synergy in Ultra-High-Performance Concrete-Optimized Prestressed Concrete Cylinder Pipes: Achieving Lightweight Design for Sustainable Infrastructure

**DOI:** 10.3390/ma18092144

**Published:** 2025-05-06

**Authors:** Yunfei Xie, Chenyang Yuan, Yajun Lv, Weifeng Bai, Yizhen Zhang

**Affiliations:** 1 School of Architecture, North China University of Water Resources and Electric Power, Zhengzhou 450045, China; xieyunfei@ncwu.edu.cn; 2 School of Water Conservancy, North China University of Water Resources and Electric Power, Zhengzhou 450045, China; yuanchenyang@ncwu.edu.cn; 3 School of International Education, North China University of Water Resources and Electric Power, Zhengzhou 116024, China; 202323207@stu.ncwu.edu.cn

**Keywords:** prestressed concrete cylinder pipes (PCCPs), ultra-high-performance concrete (UHPC), structural optimization, numerical modeling, lightweight design

## Abstract

While a large diameter is critical for maintaining water delivery efficiency in prestressed concrete cylinder pipes (PCCPs), excessive weight fundamentally limits their practical application. This study proposes a weight reduction strategy through material optimization and structural redesign. A full-scale experimental model of 2.8 m inner diameter PCCP was used to validate the finite element analysis method. Comparative numerical models were established to analyze strain/stress distribution in mortar coatings when using ultra-high-performance concrete (UHPC) versus conventional concrete cores. The key findings reveal that UHPC implementation reduces maximum coating strain by 20–30% compared to its conventional concrete counterparts. Multivariate linear regression analysis yielded a predictive formula that explicitly correlates the elastic modulus of the concrete core, core thickness, and mortar stress. This relationship permits the direct optimization of core thickness reductions according to the elastic modulus characteristics of UHPC materials. Verification through two case studies demonstrated a 25–35% core thickness reduction compared to the Chinese standard specifications while maintaining structural integrity, corresponding to an 18–22% total weight reduction. The proposed methodology successfully resolves the inherent weight limitation of conventional PCCPs while achieving equivalent hydraulic capacity, providing an effective pathway for sustainable infrastructure development through material-efficient design.

## 1. Introduction

Water is an indispensable resource for life, and ensuring its efficient and secure transportation is crucial in contemporary society. The construction and maintenance of water infrastructure has been at the forefront of civil engineering, with the development of robust and reliable pipelines as critical components. Among the various pipeline materials and designs, prestressed concrete cylinder pipes (PCCPs) have emerged as a preferred choice owing to their exceptional strength, durability, and longevity in high-pressure water conveyance systems. PCCPs [1,2], characterized by high strength, strong impermeability, and good durability, are widely used in large-scale water conservancy projects and municipal drainage infrastructure projects, such as the world’s largest PCCP pipeline project—the “Great Man River” in Libya—and China’s South-to-North Water Diversion Project. However, despite their widespread use, there are ongoing challenges related to their structural integrity, leakage prevention, and long-term performance under various environmental conditions. To address these problems, many scholars have conducted relevant research [3,4,5,6].

The main components of the PCCP structure include the prestressing wire, concrete core, mortar coating, and steel cylinder. Research has shown [7,8] that the prestressing wire in a PCCP can break or even burst in corrosive or hydrogen embrittlement environments, directly affecting the safe operation of the PCCP. Therefore, ensuring a normal working environment for prestressed steel wires has become the focus of researchers [9,10,11,12], and this is of great significance for ensuring the service life of PCCPs. References [13,14] conducted experiments and finite element analyses on the bearing capacity of pipelines after wire breakage, studied the changes in the bearing capacity of pipelines after wire breakage, and explored the influence of wire breakage on pipeline bearing performance. Their research results provide a basis for the risk assessment of pipelines after wire breakage. Zhai [15] investigated the effects of the width and thickness of a carbon fiber-reinforced polymer (CFRP) on the reinforcement effect of a new technology, namely externally bonded CFRP-reinforced PCCP. Hu et al. [16,17] utilized comprehensive testing and three-dimensional finite element analysis techniques to simulate the complex bonding interface between CFRP and concrete, and further studied the repair technology of PCCP. However, the limitations of PCCP in engineering applications are of less research interest. According to the design specification for PCCP structures [18], the maximum diameter of a PCCP can reach 4 m, the thickness of the concrete core needs to be 260 mm, and the weight per meter of the PCCP should be approximately 9.78 t. In the future, the diameter may also be increased as required. Therefore, the question of whether such a large-diameter PCCP structure can be light and thin without reducing its strength and durability deserves great attention [19].

As the main structural component of a PCCP, the concrete core is responsible for the internal pressure and external load, which ensure the pipeline’s integrity and structural stability. In general, a thicker concrete core can provide a higher bearing capacity and durability; however, it also increases the weight and cost of pipelines. A thinner concrete core can reduce the weight of the PCCP, which is particularly important for transportation and installation processes, especially when long-distance transportation or installation is required in inaccessible areas. Furthermore, a reduction in the concrete core thickness can reduce the use of concrete, thereby reducing cement consumption, hydration heat, production costs, and CO_2_ emissions, which is in line with the development trend of green and low-carbon technologies. However, thinner concrete cores may require more frequent maintenance and repair because they may be more vulnerable to damage, thereby affecting the normal working environment of the prestressed steel wires [20]. However, the use of high-performance materials such as ultra-high-performance concrete (UHPC) can improve the durability of concrete cores and reduce maintenance requirements [19]. Although the initial investment may increase owing to the use of high-performance materials, in the long run, owing to the reduction in maintenance costs and the extension of service life, the economic benefits can be increased. Therefore, it is possible to use UHPC instead of ordinary concrete in PCCP design to considerably improve the PCCP’s structural performance, economy, sustainability, and convenience of construction and maintenance [21,22].

UHPC, an innovative cement-based composite material, is famous for its excellent strength, bearing capacity, and durability [23,24,25,26,27,28,29]. These characteristics enable UHPC to significantly reduce the size of the structural components of structures and effectively reduce the weight of the overall structure. In recent years, UHPC has made significant progress in terms of theoretical research, engineering applications, standardization, and industrial development [30,31,32]. With the increasing demand for green durability, UHPC is expected to play an important role in the future of the concrete industry. According to incomplete statistics, as of the end of 2016, there were over 150 bridges in various countries around the world that used UHPC as their main structural material, and more than 400 bridges that applied UHPC materials. A bridge that uses UHPC material as its front panel is the Little Cedar Creek Bridge in Fayette County, USA [33].

In summary, UHPC has the potential to make large-diameter PCCP structures thinner and lighter without reducing their structural strength and durability; however, there is limited research on this topic. This study is structured as follows. Section 1: The application environment and research status of PCCPs, as well as the material characteristics and application characteristics of UHPC, are introduced. Section 2: A finite element analysis is conducted to verify a PCCP test model with an inside diameter of 2.8 m. Section 3: Different numerical models are established by varying the PCCP diameter, concrete core thickness, and concrete core material. Section 4: The calculation results of the numerical models are analyzed, and the influence law of the change in the concrete core material on the strain of the PCCP mortar is summarized. A method of using UHPC to reduce the thickness of the concrete core is proposed. Compared with PCCP components using ordinary concrete, the reduction in concrete core thickness results in a significant decrease in concrete usage. Section 5: A further summary of the research results is presented, and the limitations of the study and future research directions are pointed out.

## 2. Validation of Numerical Model

**Figure 1:**
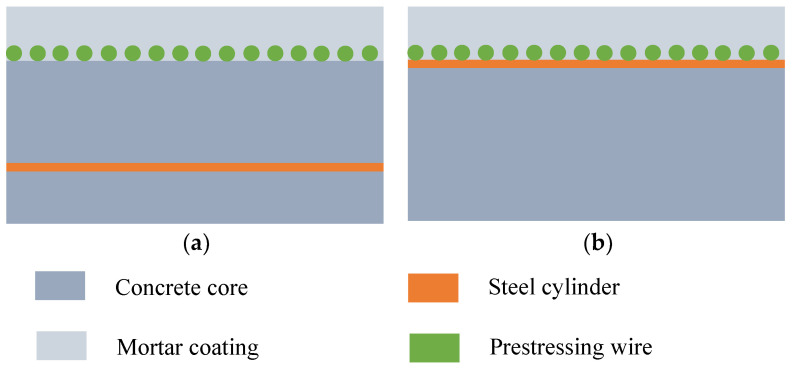
Cross-section of multilayer structure of (**a**) ECP and (**b**) LCP.

PCCP can be divided into the embedded type (ECP) and lining type (LCP) according to its structure [34]. Figure 1 shows a cross-sectional view of these two structures. Considering that the research objective of this article focuses on large-diameter PCCP, ECP is chosen as the structural form for studying pipelines. In this study, a full-scale ECP test model with an inner diameter of 2.8 m was numerically simulated and verified.

### 2.1. Element Discretization and Parameters

In the finite element model (FEM), the concrete core, mortar coating, prestressing wire, and steel cylinder were modeled separately [35]. The concrete and mortar were represented by three-dimensional eight-node hexahedral solid elements (SOLID65). The prestressing wire was represented by an annular three-dimensional double-node rod element (link180). A four-node quadrilateral shell element [36] was used to represent the steel cylinder (shell63). Considering that the winding angle of the steel wire relative to the pipe axis was approximately 90°, the contribution of the prestressing wire to the axial stress of the pipe wall can be ignored. Therefore, the continuously wound prestressing wire was considered to be a circumferential steel wire uniformly distributed on the pipe wall to simplify the model.

**Figure 2:**
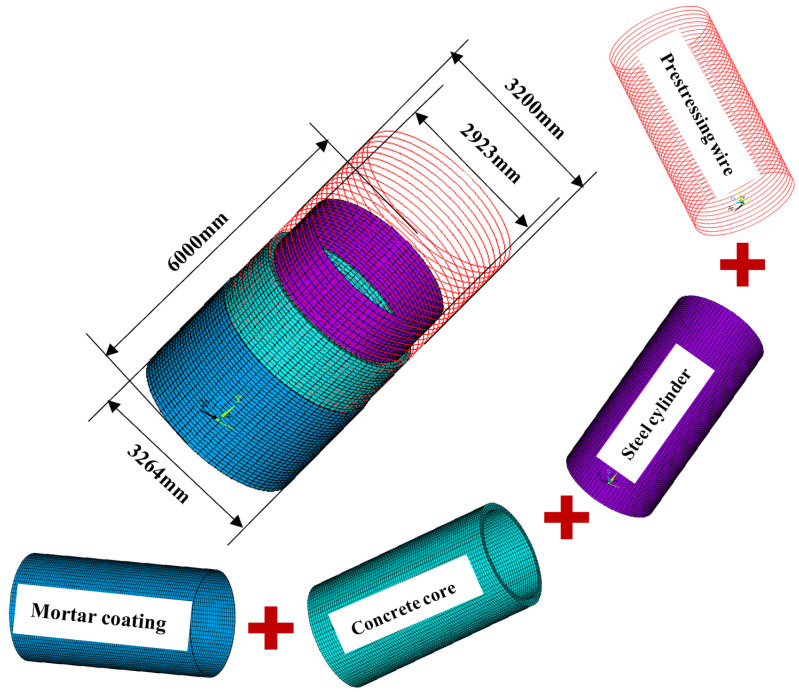
Material composition, geometric dimensions, and mesh of PCCP model (mm).

The simulation object selected in this study was a test ECP model [37]. The inside diameter of the PCCP in this test model was 2.8 m, the thickness of the concrete core was 0.2 m, the diameter of the steel cylinder was 2.923 m, the thickness of the steel cylinder was 0.0015 m, the diameter of the prestressing wire was 0.007 mm, and the spacing between the wires was 14.1 mm. The calculation and geometric parameters of the pipeline are listed in Table 1 [37]. To ensure consistency between the test and numerical models, the PCCP was placed vertically with fixed constraints at the spigot and bell ends. Figure 2 shows the geometric dimensions and grid division of the PCCP, with the grid dimensions referenced from the existing literature on grid sensitivity analysis [38,39]. The element sizes of the overall model were all less than 0.1 m, and the FEM included 34,800 nodes, 27,520 solid elements, 3520 rod elements, and 6880 shell elements. Each PCCP segment was 6 m long, with approximately 400–500 steel wires, and each ring rod unit was represented by 10 steel wires. The working internal pressure of the PCCP was 0.8 MPa, and the designed internal pressure was 1.4 times the working internal pressure, which was 1.12 MPa. The strength of the prestressing wire was *f*_su_ = 1570 MPa, and prestressing wires were applied with a wire-wrapping stress of 0.7 *f*_su_.

### 2.2. Constitutive and Contact Model

The MISO material model [40] was adopted for the concrete element, and the input data of the concrete element were obtained from the test data [41]. Considering that this study only investigated the deformation of PCCP under normal working conditions (working internal pressure of 0.8 MPa), and the limit state and failure state were not considered, the falling section in the MISO material model was assumed to be a straight line, which also met the research requirements. The protective mortar coating was simulated using a linear-elastic model. The steel cylinder was set as an ideal elastoplastic material. The constitutive relationship of the prestressed steel wire units was based on the recommendations of the American Water Works Association (AWWA) [1] and was calculated according to the following formula:

(1) $$f_{s}=\left\{\begin{array}{cc} \varepsilon_{s}E_{s} & \varepsilon_{s}\leq f_{sg}/E_{s}\\ f_{su}\left\{1-{\left[1-0.6133\left(\varepsilon_{s}E_{s}/f_{su}\right)\right]}^{2.25}\right\} & \varepsilon_{s}>f_{sg}/E_{s} \end{array}\right.$$

where *f*_su_ is the tensile strength of the steel wire, $f_{s}$ is the stress of the steel wire, *E*_s_ is the elastic modulus of the steel wire, $\varepsilon_{s}$ is the strain of the steel wire, and $f_{sg}\;$ is the wrapping stress of the steel wire.

In the numerical model, different material elements were considered for deformation coordination at the contact position. That is, elements with different material properties share nodes at the contact position without considering the relative sliding between grids [42]. To ensure consistency between the numerical model and field test results, the PCCP model was placed vertically, and the two ends of the pipe were limited only along the length and circumferential direction and not along the radial direction.

### 2.3. Analysis Procedure

**Figure 3:**
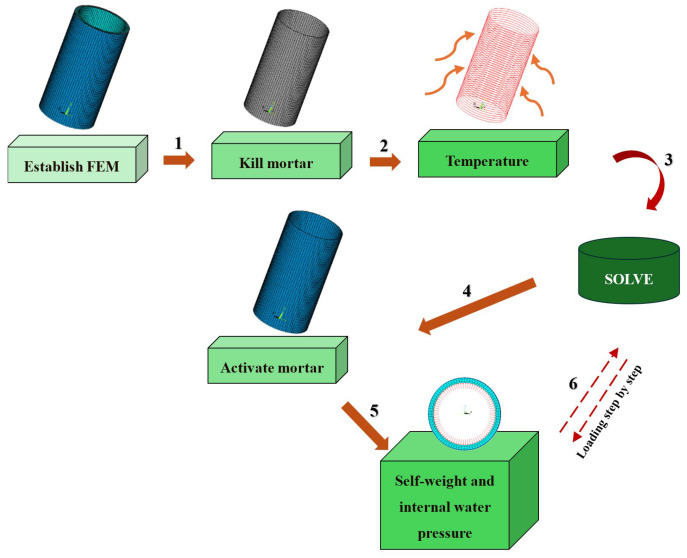
Flowchart of analysis procedure.

In the finite element calculation process, the modeling parameters were set according to the PCCP test model. The protective mortar coating was destroyed using element birth and death technology, and only the core concrete, steel cylinder, and steel wire elements were considered. Subsequently, a specified prestress was applied to the steel wires using the equivalent cooling method [15]. Next, the protective mortar coating was activated, and the self-weight of the PCCP and internal water pressure were applied consecutively. Figure 3 shows an overview of the steps.

### 2.4. Model Evaluation

**Figure 4:**
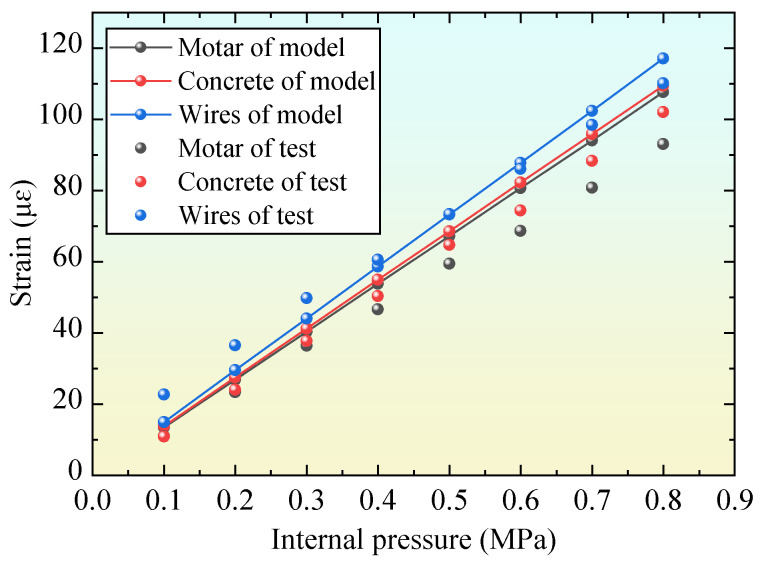
Evaluation of numerical model.

The numerical results in the same position as the PCCP (1.5 m above the bottom) were compared with the test results, as shown in Figure 4. The experimental data were obtained from the complete pipeline (that is, the PCCP was not disconnected) [37] to evaluate the FEM. The simulation results were in good agreement with the test results, as shown in the figure. The differences between the simulated and experimental values for the concrete core, steel wire, and mortar coating were 7.2%, 6.3%, and 15.7%, respectively, at an internal pressure of 0.8 MPa. During the test, the mortar was sprayed after all of the steel wires were wrapped outside the concrete, resulting in poor contact between the mortar and the concrete core. This could not achieve coordinated deformation, specifically manifested as the deformation of the mortar, which could not keep up with the deformation of the concrete core. Therefore, the test value of the mortar strain was significantly lower than the simulation value [15].

## 3. Modeling

To investigate the influence of concrete cores with different properties and thicknesses on the stress state of the PCCP mortar coating, 39 numerical models were developed using the same numerical analysis method in this study. Three of them were reference models (RM) designed according to relevant code [18] (hereinafter referred to as “code”) for PCCP models with different inside diameters (D), including parameters such as the geometric dimensions of the PCCP, the thickness of the concrete core (H_c_), and the amount of steel wire used. The other 36 models were comparative models (CM) that use UHPC as the concrete core.

The specific model design is as follows.

(1) There are three reference models (RM1, RM2, RM3), and the corresponding PCCP inside diameters are D_1_ = 4 m, D_2_ = 3.4 m, and D_3_ = 2.8 m. Ordinary concrete with a strength grade of C50 (*E_s_*_0_ = 34.5 GPa) is selected as the concrete core according to the code, and the thicknesses of the corresponding concrete cores are 260 mm, 220 mm, and 175 mm.

(2) The prestressing wire in PCCP is calculated according to the code, and the soil layer thickness is assumed to be 3 m during the calculation process.

(3) UHPC corresponding to three elastic moduli (*E_si_*, where i = 1, 2, 3) are selected for research and analysis: *E_s_*_1_ = 53 GPa, *E_s_*_2_ = 48 GPa, and *E_s_*_3_ = 45 GPa.

(4) The concrete core thickness of the comparative model is represented by *H_c_*_1_.

(5) The remaining unspecified model parameters are the same as those of the test model in the previous section.

Based on the requirements for the use of concrete in PCCP in the standard [18], the elastic modulus of UHPC selected in this article was taken from the contour map of the UHPC elastic modulus established by Ouyang [41] based on experimental data. The UHPC used in this experiment was prepared by changing the ratio between three high elastic modulus coarse aggregates, basalt, limestone, and brown corundum, based on a water–cement ratio of 0.16, a sand–cement ratio of 0.6, a water-reducing agent content of 0.5%, and a steel fiber content of 1% linear fibers and 1% long hooked-end fibers.

The parameters of the 36 comparative models are listed in Table 2. In Table 2, *H_c_* represents the thickness of the concrete core, *H_c_*_0_ represents the thickness of the ordinary concrete core, and *H_c_*_1_ represents the thickness of the high-performance concrete cores.

## 4. Calculated Results and Analysis

Steel wire breakage directly affects the safe operation of pipelines, and mortar coating is crucial for ensuring a suitable working environment for prestressed steel wires. Therefore, this section extracts the stress and strain results of mortar coatings for all models, and the results of mortar coatings for PCCP with different concrete core materials are compared.

### 4.1. Effect of UHPC on Strain of Mortar Coating

**Figure 5:**
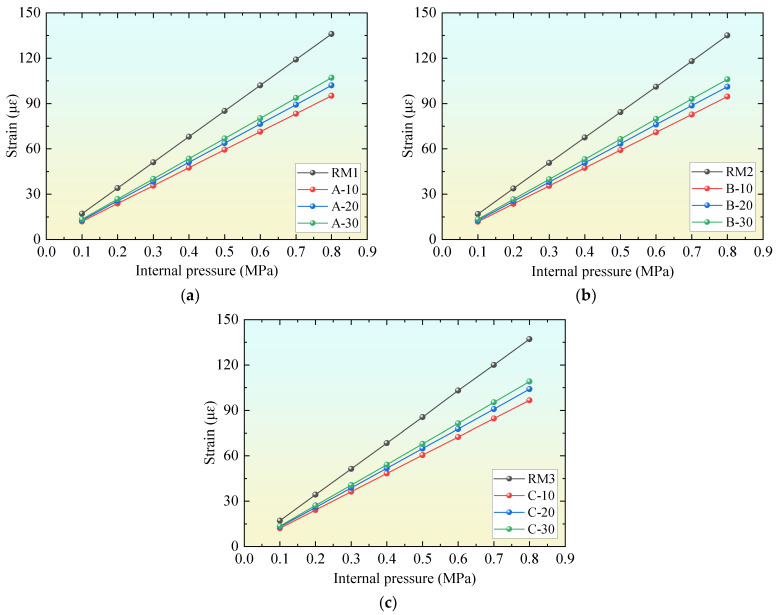
Comparison of results of PCCP designed according to specifications using ordinary concrete and UHPC: (**a**) PCCP model with diameter of 4 m; (**b**) PCCP model with diameter of 3.4 m; (**c**) PCCP model with diameter of 2.8 m.

Figure 5 shows the calculation results of the PCCP model with different inside diameters using ordinary concrete (No.: RM1, RM2, RM3) and UHPC (No.: A10, A20, A30, B10, B20, B30, C10, C20, and C30). The extracted result was located on the outer side of the mortar coating at the middle height of the PCCP model. The comparison results show that the strain of the mortar coating of the PCCP model with UHPC is less than that of the PCCP with ordinary concrete, and the decreasing amplitude increases with an increase in the elastic modulus of UHPC. Therefore, the use of UHPC in PCCP can reduce the strain of the mortar coating, improve the cracking strength of mortar, and improve the service life of the PCCP.

Table 3 shows the percentage reduction (PR) of the strain of the mortar coating of the comparison model compared with the results of the reference model when the internal pressure of the pipe is 0.8 MPa. *PR* = (*ε_RM_* − *ε_CM_*)/*ε_RM_*, where *ε_RM_* and *ε_CM_* are the strains of the mortar coating of the reference and comparison models, respectively. It was found that UHPC with the same elastic modulus has a similar influence on the mortar coating of PCCP models with different diameters. For example, when the elastic modulus was 53 GPa, the percentage reductions of the PCCP models with different inside diameters were all approximately 30%; when the elastic modulus was 48 GPa, the PRs were all approximately 25%; and when the elastic modulus was 45 GPa, the percentage reductions were all approximately 21%. These results show that the thinning efficiency of PCCP thickness is less disturbed by changes in the diameter of the concrete core and sensitive to changes in its elastic modulus. The main reason is that the mortar coating is close to the outside of the concrete core, and the deformation of the two is coordinated. After the elastic modulus of the concrete core increases, its deformation decreases, and the stress transferred to the mortar coating also decreases naturally.

### 4.2. Influence of UHPC on Thickness of Concrete Core

**Figure 6:**
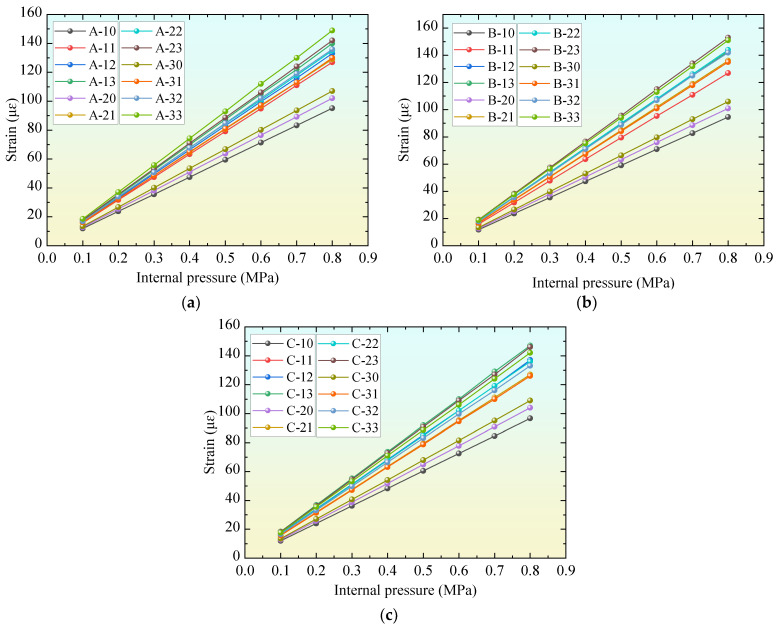
Comparison of strain of mortar coating: (**a**) results of model with D = 4 m, (**b**) results of model with D = 3.4 m, and (**c**) results of model with D = 2.8 m.

The results in the previous section show that the high elastic modulus of UHPC can reduce the strain of the mortar coating. In other words, under the same conditions, the use of UHPC can improve the safety reserve of PCCP. For this excess safety reserve, attempts can also be made to transform it into other benefits, such as a reduction in the concrete core thickness (*H_c_*). Figure 6 shows the variation in the strain of mortar coatings with the internal pressure of PCCP for all of the comparison models in Table 2.

Figure 6 shows that the reduction in *H_c_* caused an increase in the strain of the mortar coating under the same load conditions. However, comparing the results of the reference models and comparison models, the results show that the strain of the mortar coating obtained by appropriately reducing the *H_c_* of the reference model will still be lower than the result of the reference model. This means that UHPC with a high elastic modulus can transform its advantages into a reduction in concrete core thickness. This conclusion will further lead to a reduction in the volume and weight of the PCCP, which may even result in the optimization of the transportation mode of the PCCP during use.

To further investigate the relationship between the thickness of the concrete core and mortar coating, Table 4 lists the difference in stress (*σ_m_,*_∆_) between the mortar coating of the comparative model and the reference model when the internal pressure is set to 0.8 MPa. *σ_m_,*_∆_ = *σ_m,CM_* − *σ_m,RM_*, where *σ_m,RM_* is the strain of the mortar coating in the reference model, and *σ_m,CM_* is the strain of the mortar coating in the comparative model. It is found that when the elastic modulus of the concrete core is the same and the inside diameter of the PCCP is the same, only *H_c_* is taken as the independent variable, and the absolute values of *σ_m_ and _∆_* tend to decrease first and then increase. In other words, once the elastic modulus of UHPC is determined, there will be an optimal *H_c_* (*σ_m_,*_∆_ = 0) that can fully utilize the redundancy brought about by the UHPC. The result of being less than or greater than the optimal H_c_ is overdrawn or does not fully utilize the surplus brought about by the UHPC. For example, for a PCCP model with D = 4 m, when using UHPC with an elastic modulus of 53 GPa, the optimal *H_c_* can be nearly reduced by 31% from 260 mm. Comparing the results of the models with different diameters, it can also be found that the reduction in *H_c_* caused by UHPC with the same elastic modulus is similar, and it is less affected by changes in the inside diameter of the PCCP, and the change in *H_c_* with the elastic modulus of UHPC is more obvious. The main reason still lies in the coordinated deformation between the mortar coating and the concrete core. Under the same load conditions, the elastic modulus of the concrete core increases, its own deformation decreases, and the stress transmitted to the mortar coating decreases. When maintaining the stress state of the mortar coating at a constant level, concrete cores with a high elastic modulus naturally do not require too thick an *H_c_*.

### 4.3. Establishing the Optimization Formula for H_c_

**Figure 7:**
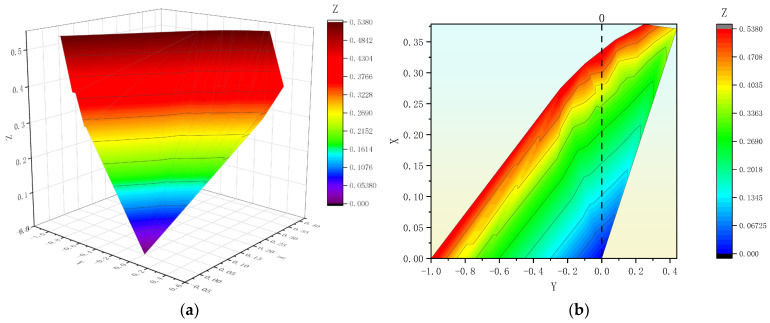
Relationship between elastic modulus and H_c_ of concrete core and stress of mortar coating: (**a**) spatial representation and (**b**) contour representation.

To describe the correlation between the elastic modulus of the concrete core and *H_c_* more accurately, this study determined the relationship between the elastic modulus of the concrete core, *H_c_*, and the stress difference of the mortar coating based on the calculation results of 39 models, as shown in Figure 7.

In Figure 7, *Z* represents the variation amplitude between the elastic moduli of the concrete cores in the reference models and comparison models at the same inside diameter of the PCCP, *Z* = *(E_si_* − *E_s_*_0_*)/E_s_*_0_; *Y* is the difference in stress between the mortar coatings of the reference model and comparison model at the same inside diameter of the PCCP, *σ_m_,*_∆_ = *σ_m,CM_* − *σ_m,RM_*; and *X* is the change rate of the concrete core thickness in the reference model and comparison model at the same inside diameter of the PCCP, *X* = *(H_c_*_0_ − *H_c_*_1_*)/H_c_*_0_. These results indicate that the graph formed by the three variables in Figure 7 appeared as an approximate spatial plane. Therefore, this study uses the multivariate linear method in MATLAB R2023b to provide the formula expression for the spatial plane in Figure 7:

*Y* = 3.0315*X* − 1.7211*Z* − 0.1181 (2)

To fully utilize the material properties of UHPC, *Y* = 0 (indicated by the dashed line in Figure 7b) can be selected as the best option in practical engineering applications. Then, based on the *E_si_* of the selected UHPC, *Z* can be obtained by substituting it into Formula (2). Finally, *H_c_*_0_ can be determined according to the code based on the inside diameter of the PCCP, which can determine the optimal *H_c_*_1_ required after using UHPC.

### 4.4. Formula Validation

Select two different inside diameters (2 m and 3.8 m) of the PCCP model as examples to verify Formula (2) and then establish the reference model and comparison model, respectively, for comparison. The elastic modulus of UHPC selected by the comparison model is taken as 48 GPa in the model with D = 2 m, and as 43 GPa in the model with D = 3.8 m. Then, *H_c_*_1_ is calculated according to Formula (2). The supplementary parameters of the example model are listed in Table 5.

**Figure 8:**
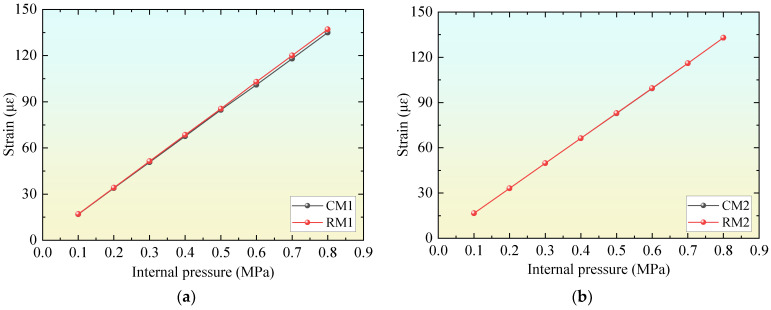
Comparison of strain of mortar coating in numerical examples: (**a**) results of example 1; (**b**) results of example 2.

Figure 8 compares the strain results of the mortar coating of the example models. The results show that the difference between the calculation results of the optimal *H_c_*_1_ model established according to Formula (2) and the model designed according to the code is less than 5%, indicating that the excess safety reserve brought about by the high-strength material characteristics of UHPC was effectively utilized and fully transformed into a reduction in the thickness of the concrete core.

## 5. Conclusions and Suggestions

This study establishes a full-size model of PCCP using the finite element method and conducts an experimental evaluation and analysis of the model. Subsequently, the application characteristics of UHPC materials in PCCP structures are investigated. By summarizing the relationship between various parameters and the results in 36 comparative models, a calculation formula for the optimal concrete core thickness of PCCP when using UHPC is given. The main conclusions are as follows:

(1) The application of UHPC as the core material in PCCP significantly reduces the strain experienced by the mortar coating. Specifically, the reduction in mortar coating strain is positively correlated with the increase in the elastic modulus of the concrete core material. Importantly, this reduction in strain remains relatively stable and is not significantly influenced by variations in the inner diameter of the PCCP. When compared to PCCP utilizing conventional concrete, UHPC demonstrates a notable enhancement in the cracking resistance. This finding is consistent with existing research [43], further validating the effectiveness of UHPC in mitigating structural deformation.

(2) Compared with the ordinary PCCP designed using the code, the thickness of the concrete core can be reduced by 20–30%, or even more with the use of UHPC, which depends on the elastic modulus of the UHPC. The greater the elastic modulus of the UHPC, the more the thickness of the concrete core can be reduced. However, considering the influence of the comprehensive factors, it is recommended that the minimum value of *H_c_* should not be less than half the recommended value in the code in practical engineering applications.

(3) The spatial relationship between the elastic modulus of UHPC and the thickness of the concrete core and the stress of the mortar layer is established, and the fitting formula of the spatial relationship diagram is given. By substituting the elastic modulus of UHPC into the formula, the optimal *H_c_* value corresponding to UHPC can be calculated directly. This formula provides a reliable calculation basis for thinning the PCCP using UHPC.

(4) In practical engineering applications, the appropriate UHPC can be selected according to the specific engineering characteristics to reduce *H_c_* and then reduce the volume and weight of PCCP. Lightweight PCCP can have a wider range of applications and provide new ideas for achieving the goal of green and low carbon.

The thinning of PCCP can improve its application range, while a reduction in concrete materials will also have more profound effects. In future research, we will focus on the correlations between more factors such as cost, life cycle, and environmental effects. In addition, the research object of this study is a single PCCP pipeline, and the influence of PCCP thinning on pipeline connections has not been considered.

## Figures and Tables

**Table 1:** Calculation material parameters and geometric dimensions of PCCP.

Material	Density (kg/m^3^)	Modulus of Elasticity (GPa)	Poisson’s Ratio	Value (mm)
Concrete core	2500	34.5	0.2	200
Prestressing wire	7850	205	0.3	7
Mortar coating	2400	24.165	0.2	32
Steel cylinder	7850	206	0.3	1.5

**Table 2:** Introduction of key parameters and 36 models.

D_1_	*E_si_*	H_c1_	NUM	D_2_	*E_si_*	H_c1_	NUM	D_3_	*E_si_*	H_c1_	NUM
4 m	E_s1_ = 53 GPa	260 mm	A-10	3.4 m	E_s1_ = 53 GPa	220 mm	B-10	2.8 m	E_s1_ = 53 GPa	175 mm	C-10
		190 mm	A-11			160 mm	B-11			130 mm	C-11
		180 mm	A-12			150 mm	B-12			120 mm	C-12
		170 mm	A-13			140 mm	B-13			110 mm	C-13
	E_s2_ = 48 GPa	260 mm	A-20		E_s2_ = 48 GPa	220 mm	B-20		E_s2_ = 48 GPa	175 mm	C-20
		200 mm	A-21			160 mm	B-21			140 mm	C-21
		190 mm	A-22			150 mm	B-22			130 mm	C-22
		180 mm	A-23			140 mm	B-23			120 mm	C-23
	E_s3_ = 45 GPa	260 mm	A-30		E_s3_ = 45 GPa	220 mm	B-30		E_s3_ = 45 GPa	175 mm	C-30
		210 mm	A-31			170 mm	B-31			150 mm	C-31
		200 mm	A-32			160 mm	B-32			140 mm	C-32
		180 mm	A-33			150 mm	B-33			130 mm	C-33

**Table 3:** The percentage reduction in the strain of the mortar coating in the comparison model compared with the reference model.

D	E_s1_	E_s2_	E_s3_
4	30.2%	25%	21.3%
3.4	29.6%	25.2%	21.5%
2.8	29.5%	24.1%	20.4%

**Table 4:** Differences in stress (*σ_m,∆_* (Pa)) between the reference models and comparison models.

H_c_	NUM	σ_m_,_∆_	H_c_	NUM	σ_m_,_∆_	H_c_	NUM	σ_m_,_∆_
260 mm	A-10	−1	220 mm	B-10	−0.97	175 mm	C-10	−0.98
190 mm	A-11	−0.24	160 mm	B-11	−0.19	130 mm	C-11	−0.24
180 mm	A-12	−0.1	150 mm	B-12	0	120 mm	C-12	−0.01
170 mm	A-13	0.08	140 mm	B-13	0.21	110 mm	C-13	0.25
260 mm	A-20	−0.84	220 mm	B-20	−0.81	175 mm	C-20	−0.8
200 mm	A-21	−0.18	160 mm	B-21	0.03	140 mm	C-21	−0.24
190 mm	A-22	−0.03	150 mm	B-22	0.22	130 mm	C-22	−0.03
180 mm	A-23	0.1	140 mm	B-23	0.44	120 mm	C-23	0.21
260 mm	A-30	−0.7	220 mm	B-30	−0.69	175 mm	C-30	−0.68
210 mm	A-31	−0.16	170 mm	B-31	0	150 mm	C-31	−0.26
200 mm	A-32	−0.03	160 mm	B-32	0.18	140 mm	C-32	−0.1
180 mm	A-33	0.3	150 mm	B-33	0.34	130 mm	C-33	0.12

**Table 5:** Supplementary parameters of example model.

	Name	D	E_s_	H_c_
Example 1	RM1	3.8 m	34.5 GPa	H_c0_ = 245 mm
	CM1	3.8 m	48 GPa	H_c1_ = 180 mm
Example 2	RM2	2 m	34.5 GPa	H_c0_ = 125 mm
	CM2	2 m	53 GPa	H_c1_ = 85 mm

## Data Availability

The datasets used and/or analyzed during the current study are available from the corresponding author upon reasonable request.

## References

[1] American Water Works Association (2019). Design of Prestressed Concrete Cylinder Pipe (AWWAC304-14).

[2] Tong D., Sun L. (2018). Research on compactness measurement method for deep backfill soil of PCCP construction. Chin. J. Undergr. Space Eng..

[3] Zarghamee M., Fok K. (1990). Analysis of prestressed concrete pipe under combined loads. J. Struct. Eng..

[4] Zarghamee M., Ojdrovic R., Dana W. (1993). Coating delamination by radial tension in prestressed concrete pipe. I: Experiments. J. Struct. Eng..

[5] Zarghamee M., Ojdrovic R., Dana W. (1993). Coating delamination by radial tension in prestressed concrete pipe. II: Analysis. J. Struct. Eng..

[6] Zarghamee M., Moharrami M. (2018). Experimental study and numerical simulation of three-edge bearing test of large diameter prestressed concrete cylinder pipes. Cond. Assess. Constr. Rehabil..

[7] Zai K., Fang H., Guo C., Li B., Wang N., Yang K., Zhang X., Du X., Di D. (2024). Using EPS and CFRP liner to strengthen prestressed concrete cylinder pipe. Constr. Build. Mater..

[8] Zarghamee M.S., Eggers D.W., Ojdrovic R., Rose B. Risk analysis of prestressed concrete cylinder pipe with broken wires. Proceedings of the Pipeline Engineering & Construction International Conference.

[9] Zhai K., Fang H., Fu B., Wang F., Hu B. (2020). Mechanical response of externally bonded CFRP on repair of PCCPs with broken wires under internal water pressure. Constr. Build Mater..

[10] Zhai K., Guo C., Fang H., Li B., Wang F. (2021). Stress distribution and mechanical response of PCCP with broken wires. Eng. Struct..

[11] Zhai K., Fang H., Guo C., Fu B., Ni P., Ma H., Fang H., Wang F. (2021). Mechanical properties of CFRP-strengthened prestressed concrete cylinder pipe based on multifield coupling. Thin-Walled Struct..

[12] Xiang T., Chen X., Guo Z., Wang J., Cui L., Qiang Y., Zhang S. (2024). Robust solid slippery surface for anti-corrosion: Experimental and simulation. Prog. Org. Coat..

[13] Zarghamee M.S. Hydrostatic pressure testing of prestressed concrete cylinder pipe with broken wires. Proceedings of the Pipeline Engineering & Construction International Conference.

[14] Liu X., Feng X. (2022). A near-wall acoustic wave-based localization method for broken wires in a large diameter PCCP using an FBG sensor array. Measurement.

[15] Zhai K., Fang H., Yang M., Sun M., Zhang X., Zhao X., Xue B., Lei J., Yao X. (2023). The impacts of CFRP widths and thicknesses on the strengthening of PCCP Failure experiment on CFRP-strengthened prestressed concrete cylinder pipe with broken wires. Structures.

[16] Hu H., Niu F., Dou T., Zhang H. (2018). Rehabilitation Effect Evaluation of CFRP-Lined Prestressed Concrete Cylinder Pipe under Combined Loads Using Numerical Simulation. Math. Probl. Eng..

[17] Hu H., Dou T., Niu F., Zhang H., Su W. (2019). Experimental and numerical study on CFRP-lined prestressed concrete cylinder pipe under internal pressure. Eng. Struct..

[18] Beijing Municipal Engineering Design and Research Institute (2011). Specification for Structural Design of Buried Prestressed Concrete Pipeline and Prestressed Concrete Cylinder Pipeline of Water Supply and Sewerage Engineering.

[19] Huawei Z. (2020). Structure Optimization Design of Prestressed Ultra High Performance Concrete Pip.

[20] Sheng Z. (2017). Study on Structural Analysis and Safety Evaluation of Large Diameter Buried Pipeline.

[21] Lv Y., Chen Y., Dai W., Yang H., Jiang L., Li K., Jin W. (2024). Preparation and properties of porous concrete based on geopolymer of red mud and yellow river sediment. Materials.

[22] Cui L., Xiang T., Hu B., Lv Y., Rong H., Liu D., Zhang S., Guo M., Lv Z., Chen D. (2024). Design of monolithic superhydrophobic concrete with excellent anti-corrosion and self-cleaning properties. Colloid Surf. A.

[23] Lv Y., Zhang W., Wu F., Wu P., Zeng W., Yang F. (2020). Static mechanical properties and mechanism of C200 ultra-high-performance concrete (UHPC) containing coarse aggregates. Sci. Eng. Compos. Mater..

[24] Liu Y., Wei Y. (2021). Effect of calcined bauxite powder or aggregate on the shrinkage properties of UHPC. Cem. Concr. Comp..

[25] Deng F., Xu L., Chi, Chen Q. (2020). Effect of steel-polypropylene hybrid fiber and coarse aggregate inclusion on the stress-strain behavior of ultra-high-performance concrete under uniaxial compression. Compos. Struct..

[26] Wu F., Xu L., Chi Y., Zeng Y., Deng F., Chen Q. (2020). Compressive and flexural properties of ultra-high-performance fiber-reinforced cementitious composite: The effect of coarse aggregate. Compos. Struct..

[27] Haile B.F., Jin D.W., Yang B., Park S., Lee H.K. (2019). Multilevel homogenization for the prediction of the mechanical properties of ultra-high-performance concrete. Constr. Build. Mater..

[28] Yoo D., Kang S., Lee J., Yoon Y.S. (2013). Effect of shrinkage reducing admixture on tensile and flexural behaviors of UHPFRC considering fiber distribution characteristics. Cem. Concr. Res..

[29] Makita T., Brühwiler E. (2014). Tensile fatigue behaviour of ultra-high performance fibre reinforced concrete (UHPFRC). Mater. Struct..

[30] Liu Y., Wei Y. (2021). Internal curing efficiency and key properties of UHPC influenced by dry or prewetted calcined bauxite aggregate with different particle size. Constr. Build. Mater..

[31] Zhang H., Ji T., Zeng X., Yang Z., Lin X., Liang Y. (2018). Mechanical behavior of ultra-high-performance concrete (UHPC) using recycled fine aggregate cured under different conditions and the mechanism based on integrated microstructural parameters. Constr. Build. Mater..

[32] Yu L., Huang L., Ding H. (2019). Rheological and mechanical properties of ultra-high-performance concrete containing fine recycled concrete aggregates. Materials.

[33] Shao X., Qiu M., Yan B., Luo J. (2017). A review on the research and application of ultra-high-performance concrete in bridge engineering around the world. Chin. J. Mater. Rep..

[34] Zhai K., Fang H., Li B., Guo C., Yang K., Du X., Du M., Wang N. (2023). Failure experiment on CFRP strengthened prestressed concrete cylinder pipe with broken wires. Tunn. Undergr. Sp. Technol..

[35] Zhai K., Fang H., Guo C., Ni P., Zhang C. (2021). Strengthening of PCCP with broken wires using prestressed CFRP. Constr. Build. Mater..

[36] Zhai K., Fang H., Guo C., Ni P., Wu H., Wang F. (2021). Full-scale experiment and numerical simulation of prestressed concrete cylinder pipe with broken wires strengthened by prestressed CFRP. Tunn. Undergr. Sp. Tech..

[37] Hu B., Fang H., Wang F., Zhai K. (2019). Full-scale test and numerical simulation study on load-carrying capacity of prestressed concrete cylinder pipe (PCCP) with broken wires under internal water pressure. Eng. Fail. Anal..

[38] Zhang W.G., Goh A.T.C. (2015). Numerical study of pillar stresses and interaction effects for twin rock caverns. Int. J. Numer. Anal. Methods Geomech..

[39] Fang H., Li B., Wang F., Wang Y., Cui C. (2018). The mechanical behaviour of drainage pipeline under traffic load before and after polymer grouting trenchless repairing. Tunn. Undergr. Sp. Technol..

[40] Thompson M.K., Thompson J.M. (2017). ANSYS Mechanical APDL for Finite Element Analysis.

[41] Ouyang X. (2022). Calculation Model and Experimental Investigation of Modulus of Elasticity in Ultra-High Performance Concrete.

[42] Zhang R., Zhang W.G., Goh A.T.C. (2018). Numerical investigation of pile responses caused by adjacent braced excavation in soft clays. Int. J. Geotech. Eng..

[43] Hajar Z., Lecointre D., Simon A. (2004). Design and construction of the world first ultra-high performance concrete road bidges. Proceedings of the International Symposium on Ultra-High Performance Concrete.

